# AMPLIFYING VOICES: DIGITAL STORYTELLING TO EXPLORE AGING IN THE RIGHT PLACE WITH HOUSING PRECARIOUS OLDER ADULTS

**DOI:** 10.1093/geroni/igae098.0951

**Published:** 2024-12-31

**Authors:** Atiya Mahmood, Sogol Haji Hosseini, Nushaiba Nanjiba, Jason Fan, Sarah Canham

**Affiliations:** Simon Fraser University, Vancouver, British Columbia, Canada; Simon Fraser University, Vancouver, British Columbia, Canada; Simon Fraser University, Vancouver, British Columbia, Canada; Simon Fraser University, Vancouver, British Columbia, Canada; University of Utah, Salt Lake City, Utah, United States

## Abstract

Digital storytelling (DS) offers an innovative qualitative approach to explore the intricate narratives surrounding aging in the right place (AIRP) for older adults with experiences of housing precarity. We engaged three participants as co-researchers to co-create a story of their life-course narratives through participation in a photovoice study followed by the development of a DS video. Study participants live in affordable rental housing with onsite supports in Metro Vancouver, Canada. Central to our DS methods is the co-creation format where our co-researchers actively shape the narrative through qualitative interviews, photographs, and video footage of the socio-spatial context of their housing highlighting the multifaceted dimensions of AIRP. The integration of photovoice photographs within the DS serves as a visual anchor, providing nuanced insights into participants’ lived experiences and aspirations regarding their current and ideal living environments. Their stories demonstrate that a combination of social, environmental, and organizational factors, as well as an interweaving of objective and subjective aspects of their housing and service experiences contribute to their stability, social integration, and well-being as they AIRP. This type of co-creative project not only serves as a means of knowledge mobilization, but also fosters dialogue and reflection between co-researchers, service providers and researchers. DS methods have the transformative potential to amplify the voices of marginalized older adults, highlighting their unique housing experiences and advocating for inclusive policy measures that prioritize their diverse needs.

